# Isolation, identification, and proposed formation mechanism of a novel hydrophilic compound formed by Maillard reaction between pyridoxamine and pentose

**DOI:** 10.1038/s41598-020-58727-8

**Published:** 2020-02-04

**Authors:** Yuri Nomi, Yuzuru Otsuka

**Affiliations:** 1 Faculty of Applied Life Sciences, Niigata University of Pharmacy and Applied Life Sciences, Niigata City, Niigata Japan; 2 Faculty of Human Life and Environmental Sciences, Ochanomizu university, Bunkyo-ku, Tokyo Japan

**Keywords:** Chromatography, Solution-state NMR

## Abstract

Pyridoxamine (PM) could competitively protect amino groups in proteins from glycating agents. Although PM is expected to react with saccharides, available data therein are limited. In this study, a novel hydrophilic compound from a model reaction solution containing PM and xylose was isolated and identified as (6a*R*,9a*R*)-1,8,9-trihydroxy-2,6a-dimethyl-6a,9a-dihydrocyclopenta[5,6]pyrano[3,4-*c*]pyridin-7(5*H*)-one with a tricyclic structure. This compound appeared to be specifically formed from pentose via 1-deoxypentosone, and its formation was facilitated over a pH range of 7.0–8.0. After heating at 90 °C for 5 h in a reaction mixture containing 30 mM PM and pentose at pH 7.4, this compound was obtained at a yield of 6.95–8.53 mM.

## Introduction

Maillard reactions between reducing sugars and amino acids, peptides, or proteins are a significant consideration in thermal food processing, storage, and cooking. This chemical reaction occurs in the body as well as in food because substrates of this reaction include amino compounds such as amino acids, peptides, or proteins, and carbonyl compounds such as reducing sugars or degradation products of carbohydrates and ascorbic acid. The reaction yields a broad array of compounds depending on reaction conditions such as pH, temperature, and the presence or absence of oxygen. In the food industry, Maillard reaction products are widely employed to modify taste, aroma, color, texture, and biological activity. However, toxic substances such as heterocyclic amines (HCAs), acrylamide, and 4(5)-methylimidazole are also produced via Maillard reactions^1–3^ as are advanced glycation end products (AGEs) in later stages of the reaction. *In vivo* AGEs are associated with increased age, and AGE accumulation is facilitated by the onset of age-related diseases and especially metabolic syndromes. Exogenous AGEs derived from the diet are thought to accumulate in the body after intestinal absorption and contribute to the development of diabetes and related complications^4^. As such, the physiological and pathological effects of this reaction have been intensively investigated.

Pyridoxamine (PM), a form of vitamin B6, is a coenzyme for enzymatic transamination *in vivo* and is found in poultry products such as chicken livers, chicken white meat, and egg yolks^5^. PM potently inhibits the formation of AGEs and protects against tissue damage induced by the chemical modification of proteins that occurs in diabetes and other diseases^6^. Several studies have indicated that PM can eliminate certain toxic carbonyl species derived from sugars and lipids, such as glyoxal, methylglyoxal, glycolaldehyde, and 1,4-dicarbonyls^7–9^. Recent reports indicated that PM could reduce amounts of 2-amino-1-methyl-6-phenylimidazo[4,5-b]pyridine (PhIP), 2-amino-3,8-dimethylimidazo[4,5-f]quinoxaline (MeIQx), and acrylamide^10,11^. This inhibitory mechanism directly traps the intermediate, preventing progress on the pathway of formation, and PM has the potential to be used to inhibit the production of toxic compounds such as HCAs and acrylamide. PM could also potentially treat chronic diseases induced by carbonyl stress. Recently, Itokawa *et al*. reported that high doses of PM decreased plasma pentosidine levels and that this treatment mitigated psychotic symptoms in some schizophrenia patients presenting with increased carbonyl stress^12^.

Thus far, many PM adducts that react with carbonyl compounds, such as degradation products from lipids and sugars, have been identified. Metz *et al*. identified several adducts formed by the incubation of PM with linoleic acid and arachidonic acid *in vitro* and showed that excretion of these adducts was elevated in the urine of PM-treated diabetic and hyperlipidemic rats over control animals^13^. Nagaraj and colleagues isolated a major reaction product of PM with methylglyoxal, identified as a methylglyoxal–pyridoxamine dimer^8^. Voziyan *et al*. showed that PM reacted rapidly with glyoxal and glycol aldehyde to form a five-ring compound with a piperazine ring at the center position^7^.

Although PM is expected to react with saccharides, published data on the reaction products of PM with saccharides are limited. Adrover and colleagues assessed the kinetics of interactions between PM and saccharides and identified multiple products of the reactions of PM with hexose and pentose^14^. 3,3′,4,4′,5,6-hexahydro-5′,6-bis(hydroxymethyl)-8′,-methylspiro[pyran-2,2′,-pyrido[4,3-e][1,3]oxazine]-3,4,5-triol, pyridoxal, and 2-[(3-hydroxy-5-(hydroxymethyl)-2-methylpyridin-4-yl)methylamino]acetic acid were isolated and identified from the PM–hexose reaction system, and 2-[(3-hydroxy-5-(hydroxymethyl)-2-methylpyridin-4-yl)methylamino]acetic acid was identified as the major product in a PM–pentose reaction model system^14^. However, these compounds were isolated using chromatography with an ODS column, which is not equipped to segregate polar compounds. Recently, several columns that can separate and retain polar compounds have become commercially available, and we have employed them to identify as yet unknown polar reaction products of PM with sugars.

This study investigated heated hydrophilic reaction products derived from PM and saccharides and found and isolated a novel hydrophilic compound formed from PM and xylose. The chemical structure and factors affecting the formation of this compound were clarified, and its formation mechanism was estimated using ^13^C-labeled experiments and analyses of α-dicarbonyls.

## Results and Discussion

### Analysis of model maillard solution of PM heated with saccharides

**Figure 1:**
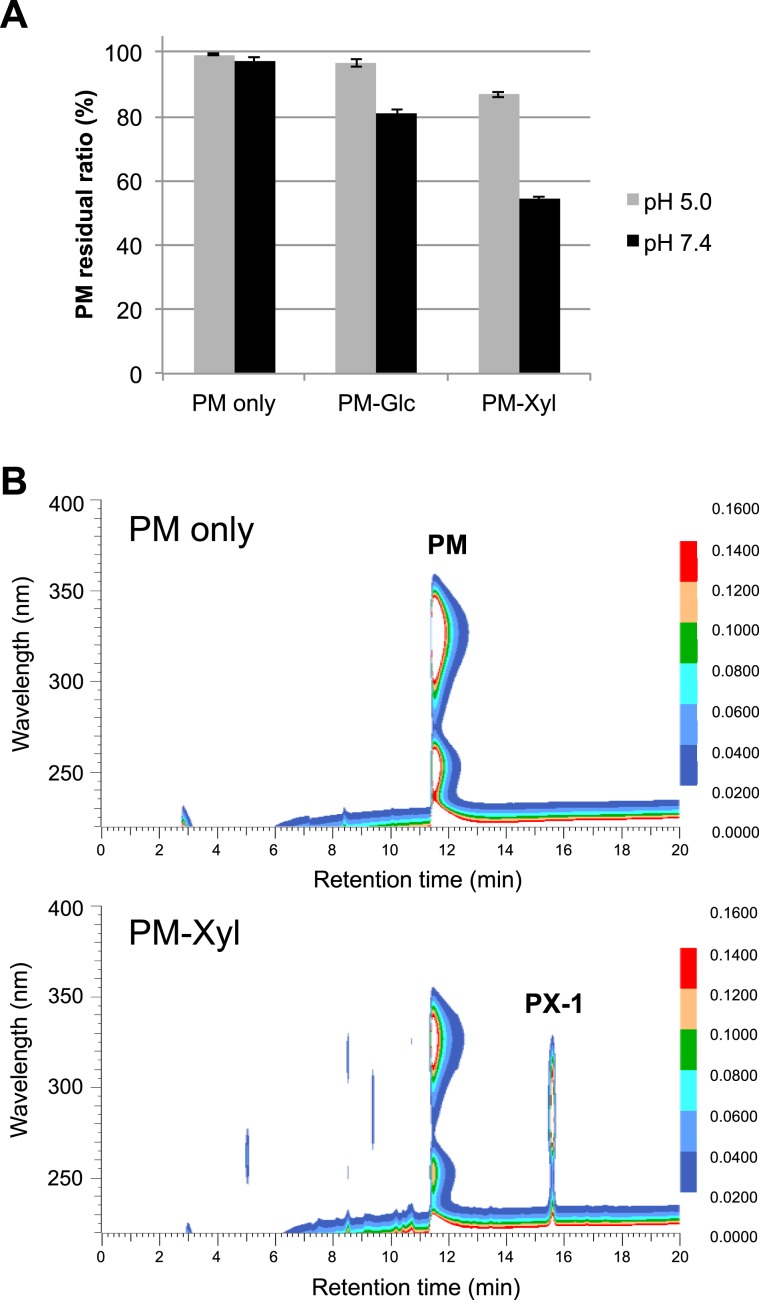
Residual ratios of PM after heating with sugars at 90 °C for 3 h (**A**) and comparison of DAD-chromatograms of the incubation mixture of PM only and PM with Xyl (**B**). PM (30 mM) with D-xylose (30 mM) or D-glucose (30 mM) was dissolved in a 500 mM sodium acetate buffer (pH 5.0) or sodium phosphate buffer (pH 7.4). These solutions were then heated to 90 °C for 3 h. A solution containing PM alone was heated simultaneously as a control. The sample was diluted 10 times with distilled water and analyzed with DAD-HPLC.

We investigated the reactivity of PM with saccharides by preparing heated solutions of PM with glucose or xylose and analyzing them with HPLC. The residual rate of PM in each heated solution is shown in Fig. 1A. PM reacted with both monosaccharides, and its reactivity with xylose was greater than that for glucose. Increasing pH values facilitated the reactions with monosaccharides, indicating that PM was consumed by the Maillard reaction. Further investigation was carried out on the solution demonstrating the highest reactivity, which was that with xylose at pH 7.4. A heated solution of PM with xylose at pH 7.4 was analyzed using DAD-HPLC to identify products formed from this reaction. Compared to the control sample without xylose, a marked decrease in PM and the formation of new reaction products were observed in the experimental sample (Fig. 1B). In the DAD-HPLC profile, one major peak (retention time 15.7 min; PX-1) was observed at absorption maxima at 280 nm. This peak was further purified using preparative HPLC, enabling 10.5 mg of purified compound to be obtained.

### Structural analysis of PX-1

Structural analysis of isolated PX-1 was performed with MS and NMR. The ESI-MS spectrum of PX-1 showed a [M + H]^+^ peak at *m/z* 264, indicating a molecular weight of 263 Da. The molecular formula of PX-1 was determined through high-resolution ESI-MS, and its mass spectrum resolved the [M + H]^+^ peak at *m/z* 264.0867 (for C_13_H_13_N_1_O_5_, 264.0872). This molecular formula corresponds to the addition of one molecule of xylose to PM and the loss of two molecules of water and one molecule of ammonia along with two hydrogen atoms (PM + xylose − 2 H_2_O − NH_3_ − 2H).

**Figure 2:**
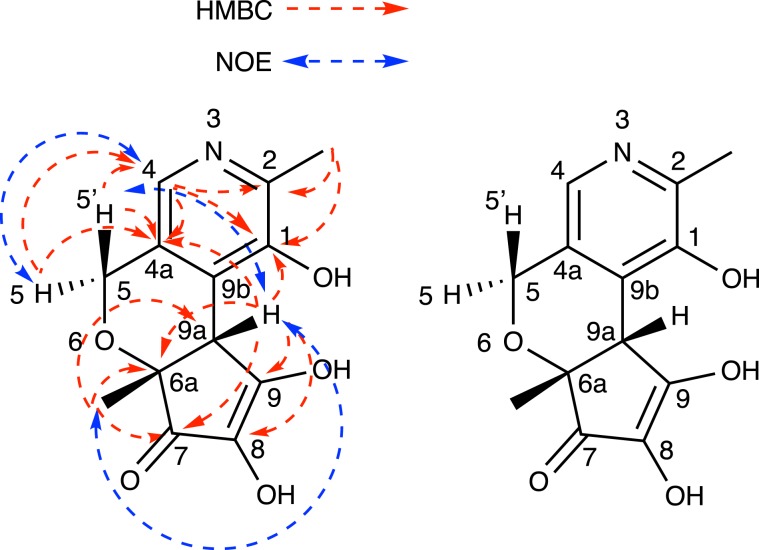
HMBC and NOESY spectral data and identified structure of PX-1. HMBC spectral data are shown as monodirectional red arrows, and NOESY data are shown as bidirectional blue arrows. Of note, it is possible that C-9 is the keto form, and C-7 is the enol form.

The ^1^H-NMR, ^13^C-NMR, heteronuclear single-quantum coherence (HSQC), and heteronuclear multiple-bond correlation (HMBC) spectra of PX-1 in DMSO-*d*_6_ were measured and compared with those of PM. Table 1 shows the ^1^H- and ^13^C-NMR data of PX-1. The comparison demonstrated that all of the ^1^H and ^13^C signals from a pyridine moiety, one methyl group, and one hydroxyl group from PM were also represented in PX-1 (C-1, C-2, C-4, C-4a, C-9b, and C-2-methyl). Conversely, the ^1^H signal of one of the methylenes (δ 59.7, C-5) shifted, from a singlet to a geminal coupled proton (δ 4.37 and 4.54, d, *J* = 12.9 Hz, H-5 and 5′), indicating the formation of an ether linkage and binding to an asymmetric carbon atom. In addition, the ^1^H signal of C-9a changed from one methylene to one methine proton (δ 3.70, H-9a). The ^13^C signals of one methyl group (δ 21.8, C-6a-methyl) and four quaternary carbons at δ 89.3 (C-6a), δ 184.3 (C-7), δ 129.7 (C-8), and δ 181.9 (C-9) were only observed in PX-1. An HMBC experiment with PX-1 supported the ^1^H-^13^C long-range couplings from H-9a (δ 3.70) to C-4a (δ 134.4), C-1 (δ 154.7), C-6a (δ 89.3), C-7 (δ 184.3), C-9 (δ 181.9), and C-8 (δ 129.7), and from H-6a-methyl (δ 1.42) to C-9a (δ 54.3), C-6a (δ 89.3), and C-7 (δ 184.3) (Fig. 2). The presence of a 2,3-dihydroxy-5-methylcyclopent-2-en-1-one structure containing C-6a, C-7, C-8, C-9, C-9a, and C-6a-methyl in PX-1 was proposed based on the molecular formula of PX-1. In addition, nuclear Overhauser effect spectroscopy (NOESY) of PX-1 showed cross peaks between δ 4.54 (H-5′) and δ 3.70 (H-9a), and between δ 3.70 (H-9a) and δ 1.42 (H-6a-methyl). Taken together, these observations enabled us to propose a structure for PX-1 (Fig. 2). PX-1 was thus identified as (6a*R*,9a*R*)-1,8,9-trihydroxy-2,6a-dimethyl-6a,9a-dihydrocyclopenta[5,6]pyrano[3,4-*c*]pyridin-7(5*H*)-one, a novel compound, according to the CAS database. Owing to its high hydrophilicity, PX-1 is not retained by the ODS columns generally used for separation. This and other hydrophilic compounds were thereby likely missed in previous studies using ODS column chromatography.

**Table 1:** 1H- and ^13^C-NMR data of PX-1.

Atom number	δ C (ppm)	δ H (ppm)	HMBC	NOESY
1	154.7		H(4), H(2-methyl), H(9a)	
2	138.9		H(4), H(2-methyl)	
2-methyl	18.6	2.23 (3 H, s)		
4	140.0	7.80 (1 H, s)	H(5), H(5′)	H(5)
4a	134.4		H(4), H(5), H(5′), H(9a)	
5	59.7	4.37 (1 H, d, 12.9)		H(4)
5′		4.54 (1 H, d, 12.9)		H(9a)
6a	89.3		H(9a), H(6a-methyl)	
6a-methyl	21.8	1.42 (3 H, s)		H(9a)
7	184.3		H(9a), H(6a-methyl)	
8	129.7		H(9a)	
9	181.9		H(9a)	
9a	54.3	3.70 (1 H, s)	H(6a-methyl)	H(5′), H(6a-methyl)
9b	133.8			

PX-1 was dissolved in DMSO-*d*_6_. These assignments were established by ^1^H–^1^H COSY, HSQC, HMBC, and NOESY experiments. Please refer to Fig. 2 for numbering.

### Formation mechanism of PX-1

**Figure 3:**
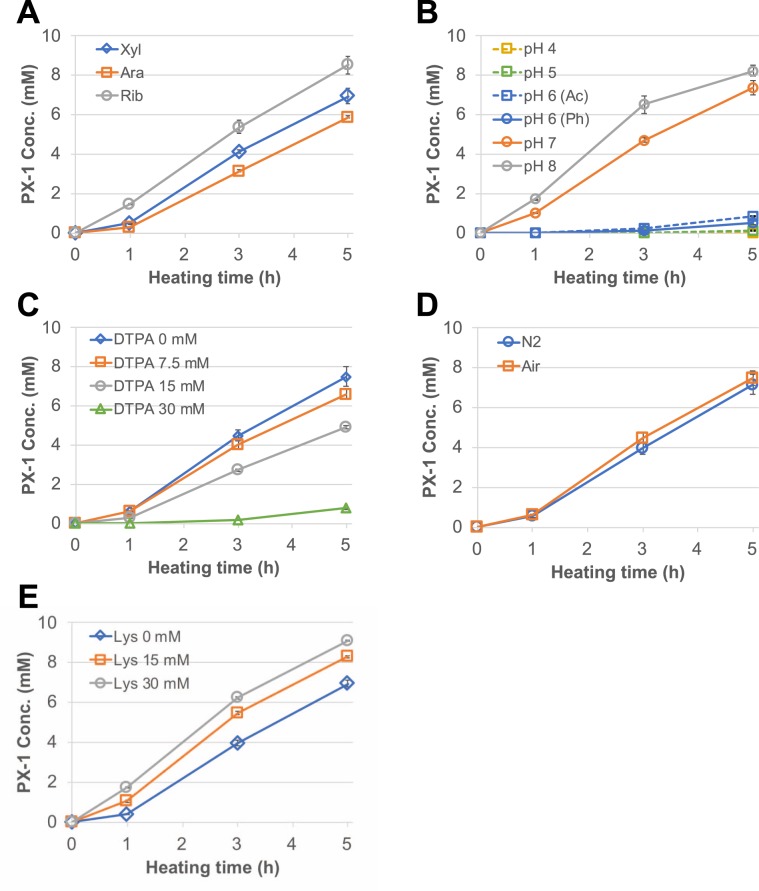
Effects of different sugars (**A**), pH (**B**), DTPA (**C**), oxygen (**D**), and lysine (**E**) on the production of PX-1. Vertical bars indicate standard deviations from the mean (n = 3).

We clarified the factors affecting the formation of PX-1 by investigating the effects of different sugars, including ribose, arabinose, glucose, fructose, and galactose, on the formation of PX-1. We predicted that PX-1 could be specifically formed from pentose since the number of carbon atoms of PX-1 (C_13_) was equal to those from PM (C_8_) plus xylose (C_5_). As expected, PX-1 was only formed from pentose and not detected in solutions containing hexose. After heating at 90 °C for 5 h in a reaction mixture containing 30 mM pyridoxamine and 30 mM pentose at pH 7.4, PX-1 was obtained at yields of 6.95 mM for xylose (23.2%), 5.88 mM for arabinose (19.6%), and 8.53 mM for ribose (28.4%) (Fig. 3A). These results indicated the rank order of reactivity for PX-1 production as ribose > xylose > arabinose and recapitulated data on the sugar reactivity of the Maillard reaction obtained by Laroque *et al*.^15^.

We next examined the effect of pH on PX-1 formation. As shown in Fig. 3B, PX-1 formation was pronounced over a pH range of 7.0–8.0. The effect of DTPA on PX-1 formation was investigated to explore the effects of a trace amount of metal ions in the buffer. Trace amounts of metal promote oxidation, and the presence of DTPA represents a non-oxidative condition, whereas the absence of DTPA indicates an oxidative condition. As shown in Fig. 3C, the addition of DTPA decreased PX-1 formation in a dose-dependent manner. We then examined the direct effects of oxygen on the formation of PX-1, and no differences in PX-1 formation were observed (Fig. 3D). We also found that PX-1 formation was enhanced by the presence of lysine in a dose-dependent manner (Fig. 3E). This result suggested that α-dicarbonyl compounds formed in the middle stages of the Maillard reaction participate in the formation of PX-1.

**Figure 4:**
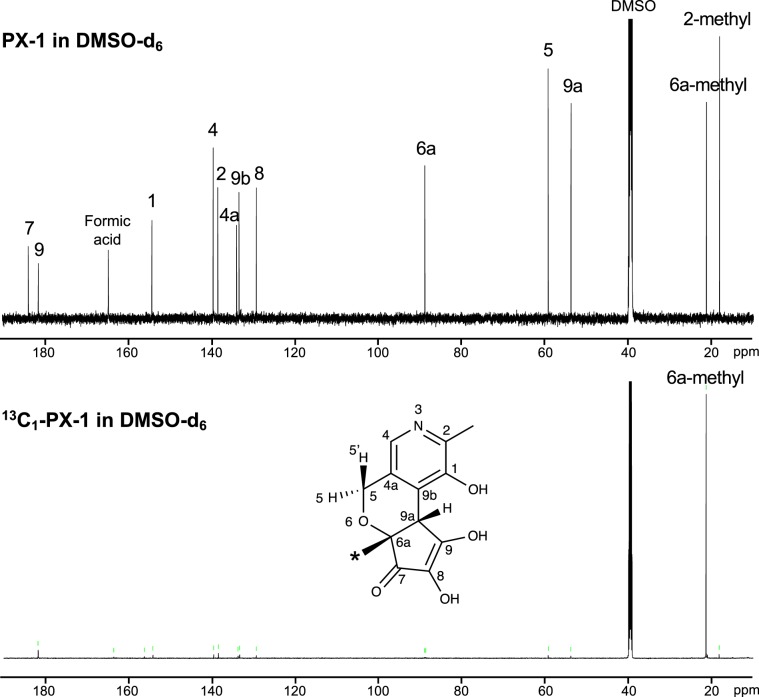
Comparison of ^13^C-NMR spectral data between PX-1 and ^13^C_1_-PX-1.

**Figure 5:**
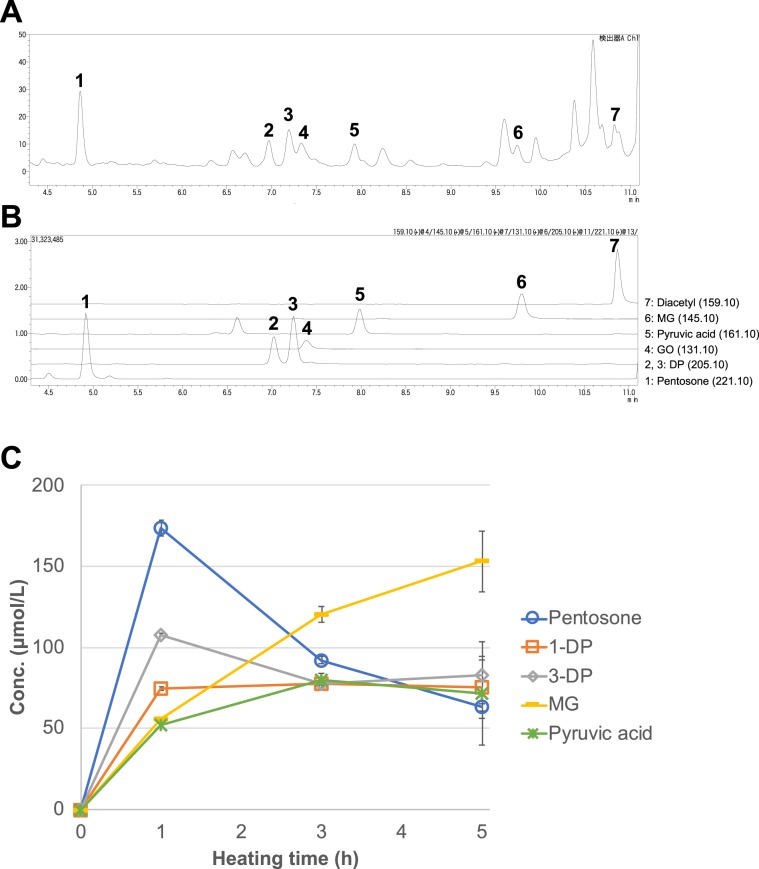
UHPLC-UV (**A**) and SIM (**B**) chromatograms, and the formation of quinoxalines (**C**) from a heated solution of PM and xylose after derivatization of α-dicarbonyl compounds by *o*-phenylenediamine, pentosone (1), 1-deoxypentosone (2), 3-deoxypentosone (3), glyoxal (4), pyruvic acid (5), methylglyoxal (6), and diacetyl (7).

We then clarified the formation mechanism of PX-1 with an isotope labeling experiment with [1-^13^C] xylose. ^13^C-labeled PX-1 was prepared and purified via the same procedure as unlabeled PX-1. The ESI-MS spectrum of ^13^C-labeled PX-1 showed an [M + H]^+^ peak at *m/z* 265. This [M + H]^+^ peak was 1 Da higher than that of unlabeled PX-1 at *m/z* 264, indicating that a single carbon atom from the C-1 of xylose was incorporated into PX-1. The ^13^C-NMR spectra of ^13^C_1_-PX-1 were measured and compared with those of unlabeled PX-1 to reveal the position where the single carbon atom (^13^C_1_) from the C-1 of xylose was incorporated and indicated that the carbon atom from the C-1 of xylose was incorporated into the C-6a-methyl of PX-1 (Fig. 4). These findings suggest that 1-deoxypentosone degraded from Amadori compounds through the generation of 2,3-endiol is involved in the formation of PX-1. In the Maillard reaction with pentose at neutral pH, 1-deoxypentosone is reported to be a major intermediate compound formed in the presence of phosphate ions^16^, and indeed, PX-1 formation was facilitated by the addition of lysine (Fig. 3E). This hypothesis was confirmed by conducting qualitative and quantitative analyses of α*-*dicarbonyls. Seven types of α*-*dicarbonyls containing 1-deoxypentosone from a heated solution of PM and xylose were identified using UV-LC-MS in selected ion monitoring (SIM) and product ion (PI) modes (Fig. 5A,B). Peaks 2 and 3 showed the same [M + H]^+^ peak at *m/z* 205.10 and were identified as 1-deoxypentosone (1-DP) and 3-deoxypentosone (3-DP) through the analysis of mass spectra in PI mode (Supplementary Fig. S1). Also, two peaks (retention times 6.6 and 8.0 min) appeared in the SIM chromatogram of *m/z* 161.10 (Fig. 5B), and peak at retention time 8.0 min was identified as pyruvic acid through the analysis of mass spectrum in PI mode (Supplementary Fig. S2). Among them, two quinoxalines derived from glyoxal (GO; **4**) and diacetyl (**7**) could not be distinguished by UV chromatogram at 316 nm. The other five quinoxalines, derived from pentosone (**1**), 1-DP (**2**), 3-DP (**3**), pyruvic acid (**5**), and methylglyoxal (MG; **6**), were quantitated. As shown in Fig. 5C, 1-DP was detected after 1 h of heating, and its concentration remained unchanged during the subsequent 4 h of heating. This phenomenon suggests that 1-DP was rapidly consumed by PM to form PX-1.

**Figure 6:**
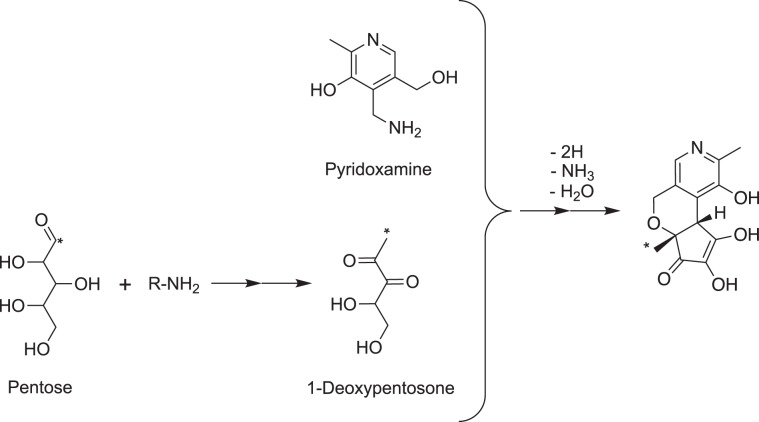
Suggested formation schematic of PX-1.

A plausible formation scheme of PX-1 based on the above results is shown in Fig. 6. PX-1 is likely formed via the 1-DP degraded from Amadori compounds. The formation of PX-1 may decrease the amount of short-chained dicarbonyl compounds, such as methylglyoxal and diacetyl, degraded from 1-DP.

## Conclusion

In this study, a novel Maillard reaction product was isolated and identified as the major product of a heated reaction of PM with xylose, and a potential formation mechanism of PX-1 is provided. Further research on the existence of PX-1 in food and biological samples and its functional properties will be needed in the future.

## Materials and Methods

### Reagents

All chemical and chromatographic reagents used were of HPLC or LC/MS grade. Pyridoxamine dihydrochloride monohydrate was obtained from Tokyo Chemical Industry (Tokyo, Japan). D(+)-Xylose, L(+)-lysine monohydrochloride, acetonitrile (ACN), and 1 M ammonium formate solution were obtained from Wako Pure Chemical Industry (Osaka, Japan). D-[1-^13^C]xylose (99%) was obtained from Cambridge Isotope Laboratories, Inc. (MA, USA). Other reagents were obtained from Kanto Kagaku (Tokyo, Japan).

### Incubation of PM with saccharides and analysis by DAD-HPLC

PM (30 mM) with D-xylose (30 mM) or D-glucose (30 mM) was dissolved in a 500 mM sodium acetate buffer at pH 5.0 or 500 mM sodium phosphate buffer at pH 7.4. This solution was transferred to a test tube with a screw cap that was then heated to 90 °C for 3 h. A solution containing PM alone was heated simultaneously as a control. The sample was diluted 10 times with distilled water and analyzed by a diode array detection (DAD)-HPLC equipped with a 250 mm × 4.6 mm i.d. Scherzo SM-C18 column (Imtakt Co., Ltd., Kyoto, Japan). Mobile phases consisted of solvent A containing 5 mM ammonium formate in water and solvent B containing 150 mM ammonium formate in water/ACN (30/70, v/v). Separation conditions were a linear gradient from 0% to 100% of solvent B over 45 min at 40 °C. The flow rate was 1.0 mL/min. The diode array detector was set at 220–500 nm, and 10 µL of the sample was injected. The peak area of residual PM (retention time 11.5 min) was quantitated by an external standard method at a wavelength of 320 nm.

### Isolation of PX-1 from a reaction mixture of PM and xylose

PM (60 mM) and D-xylose (60 mM) were dissolved in 5 mL of sodium phosphate buffer (500 mM, pH 7.4). This solution was transferred to a test tube with a screw cap, which was then heated to 90 °C for 5 h. After cooling, this solution was frozen at −20 °C before injection into a preparative HPLC. The PX-1 in this solution was purified by a preparative HPLC equipped with a 250 mm × 10 mm i.d. Scherzo SM-C18 column (Imtakt Co., Ltd., Kyoto, Japan). Mobile phases consisted of solvent A containing 5 mM ammonium formate in water and solvent B containing 150 mM ammonium formate in water/ACN (30/70, v/v). The separation conditions were a linear gradient from 2% to 50% of solvent B over 20 min at 40 °C. The flow rate was 3.0 mL/min. The diode array detector was set at 220–500 nm, and 70 µL of the sample was injected repeatedly until sufficiently pure samples were obtained (3.5 mL total injected volume). The peak at retention time 16.5 min was collected. The sample was lyophilized, and a pure component (10.5 mg) was obtained.

### Structural analysis

Electrospray ionization (ESI) mass spectra were measured using the AB SCIEX QTRAP^®^ and TripleTOF^®^ 4600 systems (AB SCIEX, Tokyo, Japan). The ^1^H-/^13^C-NMR spectra were measured using a Bruker Avance III 600 spectrometer (Bruker BioSpin, Karlsruhe, Germany), and chemical shifts were given in δ values relative to that of the solvent (dimethylsulfoxide-*d*_6_; δ_H_ 2.49, δ_C_ 39.7) on a tetramethylsilane scale.

### Preparation of model solutions under different conditions

PM (30 mM) and 30 mM of one of six sugars, namely, xylose, ribose, arabinose, glucose, fructose, and galactose, were dissolved in a 200 mM sodium phosphate buffer at pH 7.4 to assess the effects of different types of sugars on PX-1 formation. PM (30 mM) and xylose (30 mM) were dissolved in a 200 mM sodium acetate (pH 4.0, 5.0, or 6.0) or 200 mM phosphate buffer (pH 6.0, 7.0, or 8.0) to assess the effects of pH on formation. PM (30 mM) and xylose (30 mM) with or without diethylenetriamine pentaacetic acid (DTPA; 7.5, 15, and 30 mM) were dissolved in a sodium phosphate buffer (200 mM, pH 7.4) to assess the effect of a chelator. PM (30 mM) and xylose (30 mM) with or without nitrogen gas bubbling were dissolved in a sodium phosphate buffer (200 mM, pH 7.4) to assess the effect of adding oxygen. PM (30 mM) and xylose (30 mM) with or without lysine (Lys; 0, 15, and 30 mM) were dissolved in a sodium phosphate buffer (200 mM, pH 7.4) to assess the effect of adding an amino compound. These mixtures were transferred to test tubes with screw caps and heated to 90 °C for 5 h. After heating, the model solutions were cooled and subjected to HPLC for quantitation of PX-1.

### Quantitation of PX-1 contained in model solutions

The model solutions were analyzed by diode array detection (DAD)-HPLC equipped with a CAPCELL PAK ADME S5 column (250 mm × 4.6 mm i.d.; Shiseido, Tokyo, Japan). Mobile phases consisted of solvent A containing 10 mM ammonium formate in water and solvent B containing ACN. The separation conditions were a linear gradient from 2% to 20% of solvent B over 20 min at 40 °C. The flow rate was 1.0 mL/min. The diode array detector was set at 200–600 nm, and 10 µL of a sample was injected. PX-1 was eluted at a retention time of 16.5 min and quantitated by an external standard method at a wavelength of 280 nm.

### Preparation of ^13^C-labeled PX-1 from the reaction mixture of PM and [1-^13^C] xylose

^13^C-labeled PX-1 was prepared in a manner similar to that used for the isolation of its unlabeled counterpart, except that D-[1-^13^C] xylose was utilized instead of D-xylose. The entire sample was injected into a preparative HPLC system operating under the same conditions as those for unlabeled PX-1. The peak corresponding to ^13^C-labeled PX-1 (retention time 16.5 min) was collected. The sample was lyophilized, and a pure component (6.9 mg) was obtained. Structural analyses were performed with MS and NMR.

### Quantitation of quinoxalines derived from α-dicarbonyls

#### Derivatization procedure for the analysis of α-dicarbonyls

Analysis of α-dicarbonyl compounds was carried out according to a previously reported method with slight modifications^17^. Briefly, the model solution was prepared by dissolving PM (30 mM) and xylose (30 mM) in a 200 mM phosphate buffer at pH 7.4. These mixtures were transferred to test tubes with screw caps and then heated to 95 °C for 5 h. 500 µL aliquots of were sampled from the heated solution at 1, 3, and 5 h and then mixed with 200 μL of 0.5 M sodium phosphate buffer (pH 7.0) and 200 µL of 0.2% (w/v) *o*-phenylenediamine solution containing 18.5 mM DTPA. The mixture was kept in the dark overnight and filtered through a 0.22 µm pore membrane before UHPLC-ESI-MS analysis.

### UHPLC-ESI-MS analysis of quinoxalines derived from α-dicarbonyls

The UHPLC-MS system consisted of a Shimadzu LCMS-8030 quadrupole mass spectrometer and a Shimadzu Prominence^®^ LC-30A HPLC system (Shimadzu, Kyoto, Japan). Chromatographic separation was achieved in a Waters ACQUITY UPLC^®^ BEH Phenyl column (1.7 μm, 100 mm × 2.1 mm i.d.). The mobile phase consisted of solvent A containing 0.1% (v/v) formic acid in water and solvent B containing methanol. The separation conditions were in linear gradients as follows: from 3% to 25% of solvent B over 8.5 min, 25% to 50% of solvent B from 8.5–10 min, 50% to 80% of solvent B from 10–10.1 min, 80% of solvent B from 10.1–12 min, 80% to 95% of solvent B from 12–12.1 min, 95% of solvent B from 12.1–15 min, 95% to 3% of solvent B from 15–15.1 min, and 3% of solvent B from 15.1–25 min. A valve system was used to inject to MS from LC for 3.5–13 min. The flow rate was 0.4 mL/min. The column temperature was set at 55 °C, and 5 μL of a derivatized sample was injected. Quinoxalines were detected at UV 316 nm and quantitated by an external standard method using a calibration curve prepared with quinoxaline of 3-deoxyglucosone (3-DG) over a concentration range of 9.95–77.16 μmol/L because other α*-*dicarbonyls are not commercially available. Quinoxalines of α*-*dicarbonyls excepting 3-DG were identified through LC-MS/MS analysis and by comparing retention times and mass spectra with those reported previously^18,19^. A Shimadzu LCMS-8030 quadrupole mass spectrometer equipped with an ESI source in a positive ion mode was operated under the following conditions: nebulizing gas and drying gas with nitrogen were set at flow rates of 3.0 and 15.0 L/min, respectively, the interface voltage was set to 4.5 kV, and desolvation line and heat block temperatures were set at 300 °C and 500 °C, respectively. The mass spectrometer was used in SIM and PI modes with argon as the collision-induced dissociation gas at a pressure of 230 kPa, and the detector voltage was set to 1.78 kV. The *m/z* values of quinoxalines from α-dicarbonyls with SIM mode were set as follows: pentosone, 221.10; deoxypentosone (DP), 205.10; pyruvic acid, 161.10; glyoxal (GO), 131.10; methylglyoxal (MG), 145.10; and diacetyl, 159.10. In PI mode, the *m/z* values of the precursor ion were set at these same values. The collision energy in PI mode was set at 35 eV, and the mass spectra were measured over a range of *m/z* of 50–250.

## Supplementary information

Supplementary Information.

## Data Availability

The datasets generated and/or analyzed during the current study are available from the corresponding author on reasonable request.

## References

[1] Skog KI, Johansson MA, Jagerstad MI (1998). Carcinogenic heterocyclic amines in model systems and cooked foods: a review on formation, occurrence and intake. Food Chem. Toxicol..

[2] Moon J-K, Shibamoto T (2011). Formation of Carcinogenic 4(5)-Methylimidazole in Maillard Reaction Systems. J. Agric. Food Chem..

[3] Mottram DS, Wedzicha BL, Dodson AT (2002). Food chemistry: Acrylamide is formed in the Maillard reaction. Nature.

[4] Uribarri J (2005). Diet-derived advanced glycation end products are major contributors to the body’s AGE pool and induce inflammation in healthy subjects. Ann. N. Y. Acad. Sci..

[5] Thi Viet DH, Ide Y, Mugo AN, Yagi T (2012). All-enzymatic HPLC method for determination of individual and total contents of vitamin B6 in foods. Food & Nutrition Research.

[6] Voziyan PA, Hudson BG (2005). Pyridoxamine as a multifunctional pharmaceutical: targeting pathogenic glycation and oxidative damage. CMLS, Cell. Mol. Life Sci..

[7] Voziyan PA, Metz TO, Baynes JW, Hudson BG (2002). A Post-Amadori Inhibitor Pyridoxamine Also Inhibits Chemical Modification of Proteins by Scavenging Carbonyl Intermediates of Carbohydrate and Lipid Degradation. J. Biol. Chem..

[8] Nagaraj RH (2002). Effect of pyridoxamine on chemical modification of proteins by carbonyls in diabetic rats: characterization of a major product from the reaction of pyridoxamine and methylglyoxal. Arch. Biochem. Biophys..

[9] Amarnath V, Amarnath K, Amarnath K, Davies S, Roberts LJ (2004). Pyridoxamine:  An Extremely Potent Scavenger of 1,4-Dicarbonyls. Chem. Res. Toxicol..

[10] Wong D, Cheng K-W, Wang M (2012). Inhibition of heterocyclic amine formation by water-soluble vitamins in Maillard reaction model systems and beef patties. Food Chem..

[11] Arribas-Lorenzo G, Pintado-Sierra M, Morales FJ (2011). Isolation and Structural Characterization of Acrylamide−Pyridoxamine Adducts. Chem. Res. Toxicol..

[12] Itokawa M (2018). Pyridoxamine: A novel treatment for schizophrenia with enhanced carbonyl stress. Psychiatry Clin. Neurosci..

[13] Metz TO, Alderson NL, Chachich ME, Thorpe SR, Baynes JW (2003). Pyridoxamine traps intermediates in lipid peroxidation reactions *in vivo*: evidence on the role of lipids in chemical modification of protein and development of diabetic complications. J. Biol. Chem..

[14] Adrover M, Vilanova B, Muñoz F, Donoso J (2007). Pyridoxamine, a scavenger agent of carbohydrates. Int. J. Chem. Kinet..

[15] Laroque D (2008). Kinetic study on the Maillard reaction. Consideration of sugar reactivity. Food Chem..

[16] Rizzi GP (2004). Role of Phosphate and Carboxylate Ions in Maillard Browning. J. Agric. Food Chem..

[17] Degen J, Hellwig M, Henle T (2012). 1,2-Dicarbonyl Compounds in Commonly Consumed Foods. J. Agric. Food Chem..

[18] Kocadağlı T, Gökmen V (2014). Investigation of α-Dicarbonyl Compounds in Baby Foods by High-Performance Liquid Chromatography Coupled with Electrospray Ionization Mass Spectrometry. J. Agric. Food Chem..

[19] Henning C, Liehr K, Girndt M, Ulrich C, Glomb MA (2014). Extending the Spectrum of α-Dicarbonyl Compounds *in Vivo*. J. Biol. Chem..

