# Supplementary material for: Isolation, identification, and proposed formation mechanism of a novel hydrophilic compound formed by Maillard reaction between pyridoxamine and pentose
Source: Sci Rep. 2020 Feb 4;10:1823. doi: 10.1038/s41598-020-58727-8 (PMC7000833; doi:10.1038/s41598-020-58727-8)
Supplement: Supplementary file 1 — Supplementary Information. [file 41598_2020_58727_MOESM1_ESM.docx]

**Isolation, identification, and proposed formation mechanism of a novel hydrophilic compound formed by Maillard reaction between pyridoxamine and pentose**

Yuri Nomi^†, *^ and Yuzuru Otsuka^‡^

^†^Faculty of Applied Life Sciences, Niigata University of Pharmacy and Applied Life Sciences, Niigata City, Niigata, Japan. E-mail: ynomi@nupals.ac.jp

^‡^Faculty of Human Life and Environmental Sciences, Ochanomizu university, Bunkyo-ku, Tokyo, Japan. E-mail: otsuka.yuzuru@ocha.ac.jp

^*^Correspondence author: Yuri Nomi, Faculty of Applied Life Sciences, Niigata University of Pharmacy and Applied Life Sciences, 265-1 Higashijima, Akiha-ku, Niigata City, Niigata 956-8603, Japan. Tel: +81-250-28-5348. E-mail: ynomi@nupals.ac.jp

**Appendices**

**Fig. S1.**

The spectra of product ion scans of quinoxalines from 1-deoxypentosone (2) and 3-deoxypentosone (3) after derivatization of α*-*dicarbonyl compounds by *o*-phenylenediamine. The fragment ion peaks in accord with previously published data ^19^ shows blue arrows.

**Fig. S2.**

The spectra of SIM and product ion scans of quinoxaline from pyruvic acid (5) after derivatization of α-dicarbonyl compounds by *o*-phenylenediamine. The fragment ion peaks in accord with previously published data ^19^ shows blue arrows.
